# Probucol attenuates hyperoxia-induced lung injury in mice

**DOI:** 10.1371/journal.pone.0175129

**Published:** 2017-04-06

**Authors:** Tomonobu Kawaguchi, Toyoshi Yanagihara, Tetsuya Yokoyama, Saiko Suetsugu-Ogata, Naoki Hamada, Chika Harada-Ikeda, Kunihiro Suzuki, Takashige Maeyama, Kazuyoshi Kuwano, Yoichi Nakanishi

**Affiliations:** 1 Research Institute for Diseases of the Chest, Graduate School of Medical Sciences, Kyushu University, Fukuoka, Japan; 2 Division of Respiratory Diseases, Department of Internal Medicine, Jikei University School of Medicine, Tokyo, Japan; University of Alabama at Birmingham, UNITED STATES

## Abstract

Hyperoxic lung injury is pathologically characterized by alveolar edema, interlobular septal edema, hyaline membrane disease, lung inflammation, and alveolar hemorrhage. Although the precise mechanism by which hyperoxia causes lung injury is not well defined, oxidative stress, epithelial cell death, and proinflammatory cytokines are thought to be involved. Probucol—a commercially available drug for treating hypercholesterolemia—has been suggested to have antioxidant and antiapoptotic effects. This study aimed to assess whether probucol could attenuate hyperoxic lung injury in mice. Mice were exposed to 95% O_2_ for 72 h, with or without pre-treatment with 130 μg/kg probucol intratracheally. Probucol treatment significantly decreased both the number of inflammatory cells in the bronchoalveolar lavage fluid and the degree of lung injury in hyperoxia-exposed mice. Probucol treatment reduced the number of cells positive for 8-hydroxyl-2′-deoxyguanosine or terminal deoxynucleotidyl transferase dUTP nick end labeling (TUNEL) and suppressed NF-κB activation, Bax expression, and caspase-9 activation in lung tissues from hyperoxia-exposed mice. These results suggest that probucol can reduce oxidative DNA damage, apoptotic cell death, and inflammation in lung tissues. Intratracheal administration of probucol may be a novel treatment for lung diseases induced by oxidative stress, such as hyperoxic lung injury and acute respiratory distress syndrome.

## Introduction

Oxygen therapy is required for patients with hypoxemia to prevent serious pulmonary or cardiac disorders. However, prolonged exposure to high levels of oxygen can result in higher intensive care unit mortality [1] and may lead to acute respiratory distress syndrome (ARDS). Animal models of hyperoxic lung injury are pathologically characterized by alveolar and interlobular septal edema, hyaline membrane disease, inflammatory cell infiltration, and alveolar hemorrhage [2]. Hyperoxic lung injury in experimental animals is regarded as an established model of ARDS [3].

The pathogenesis of hyperoxic lung injury is not well defined. However, some studies demonstrated that exposure to a hyperoxic environment induced the production of reactive oxygen species (ROS), such as hydrogen peroxide, superoxide anion, and proinflammatory cytokines [4]. ROS activate extracellular signal-regulated kinases (ERK) 1 and 2, but not c-Jun N-terminal protein kinase (JNK1/2) or p38 mitogen-activated protein kinase, and induce lung epithelial cell death by apoptosis or necrosis [5,6]. The degree of apoptotic cell death induced by hyperoxia was well correlated with the severity of lung injury [7]. Exposure to hyperoxia resulted in Bax activation at the mitochondrial membrane and subsequent cytochrome c release, demonstrating that ROS acted as upstream signaling molecules that initiated cell death [8]. This shows that apoptotic signaling plays an important role in hyperoxia-induced cell death, regardless of cell death phenotype [9]. As such, ROS generation and subsequent epithelial cell death are thought to be the major causes of hyperoxic lung injury.

Probucol is a diphenolic compound with antiatherosclerotic attributes: it has low-density lipoprotein cholesterol-lowering, antioxidant, and anti-inflammatory properties. Probucol can also lower serum high-density lipoprotein cholesterol, and had thus been limited in clinical application to only a few countries, including Japan. However, the recently reported effects of probucol in patients with heterozygous familial hypercholesterolemia from a long-term follow-up study has reignited interest in this drug [10]. Another recent study showed that probucol therapy improved long-term survival after complete revascularization; i.e., percutaneous coronary intervention and bypass surgery [11]. Long-term outcomes of polymer-free sirolimus- and probucol-eluting stents were similar to those of polymer-based zotarolimus-eluting stents [12]. An antioxidant effect is the trapping of free radicals and reduction of oxidative stress by inhibiting reduced nicotinamide adenine dinucleotide phosphate (NADPH) oxidase activity [13]. Probucol also prevented endothelial-cell apoptosis induced through oxidative stress by suppressing NF-κB activation [14]. Although the precise mechanisms involved are uncertain, oxidative stress and cell death are involved in hyperoxic lung injury. Therefore, we investigated whether probucol has antioxidant and antiapoptotic effects that could protect tissues against hyperoxic lung injury in mice.

## Materials and methods

### Animals and hyperoxia exposure

The experiments were approved by the Committee on Ethics regarding Animal Experiments of Kyushu University (No. A23-196-0) and were conducted in accordance with the guidelines of the Animal Care and Use Committee, Kyushu University. Seven-week-old female C57BL/6 mice were purchased from Japan SLC Inc. (Shizuoka Japan) and housed under pathogen-free conditions for one week before the experiments. Mice were divided into four experimental groups: room-air-exposed (*n* = 10), room-air-exposed with probucol treatment (*n* = 10), hyperoxia-exposed (*n* = 10), and hyperoxia-exposed with probucol treatment (*n* = 10). Before exposure to hyperoxia or room air, mice were anesthetized with an intraperitoneal injection of ketamine hydrochloride (Daiichi Sankyo, Tokyo, Japan) and xylazine hydrochloride (Sigma-Aldrich, MO, USA). Probucol was dissolved in 100% dimethyl sulfoxide (DMSO) and then diluted 1:1000 with distilled water (final concentration of DMSO was 0.1%). The anesthetized mice received an intratracheal dose of 130 μg/kg probucol (50 μL) (Daiichi Sankyo, Tokyo, Japan), or 0.1% DMSO, from an IA-1C MicroSprayer (Penn Century, USA). On the next day, mice were exposed to room air or hyperoxia (95% O_2_) in a sealed Plexiglas chamber (Physio-Tech, Tokyo, Japan). Under exposure to hyperoxia, mice had free access to food and water. The condition of the mice was checked every two hours during the light period. No serious illness was observed that would necessitate the use of analgesics, and no mice died before the experimental points. Oxygen concentration was continuously monitored with PRO-OX (BioSpherix, NY, USA). After exposure for 72 h, mice were anesthetized with pentobarbital and hemorrhaged by opening the abdomen and cutting the descending aorta. Half of the mice in each group were used for histological evaluation and western blot analysis of the whole lung, and the others were used for bronchoalveolar lavage (BAL) fluid (BALF) analysis. This procedure was repeated twice. Samples of the left lung tissues were frozen at −80°C for western blot analysis, and right lung tissues were fixed in 10% buffered formalin. The time of exposure to hyperoxia and the concentration of oxygen were determined according to previous studies [15].

### Cell count in BALF

A tracheotomy was performed in the sacrificed mice. After insertion of the tracheal tube, the whole lung was lavaged two times with 1-ml sterile saline at room temperature. The recovered fluids were filtered through a single layer of gauze to remove mucus. Cells in the BALF were counted using a hemocytometer. Differential counts of BAL cells were performed on 200 cells stained with Diff-Quick (Baxter Diagnostics, Düdingen, Switzerland).

### Measurement of total protein content in BALF

Total protein concentration in BALF samples was measured using the Bradford protein assay (Biorad, Hercules, CA, USA) according to the manufacturer’s instructions. OD readings of samples were converted to μg/ml using values obtained from a standard curve generated with serial dilutions of bovine serum albumin (125–2000 μg/ml).

### Measurement of IL-6 level in BALF

BALF was centrifuged and the supernatant was stored at −80°C until use. Interleukin (IL)-6 level in BALF was measured with a cytokine-specific ELISA kit purchased from Biosource International (Camarillo, CA, USA). The minimum detectable dose of IL-6 was 3 pg/ml.

### Histopathology of lung tissues

After thoracotomy, the pulmonary circulation was flushed with saline, and the lungs were excised. The lung samples were fixed in 10% buffered formalin overnight and then embedded in paraffin. A 3-μm paraffin section was adhered to slides and stained with hematoxylin and eosin. The pathological grade of lung injury in the whole area of the mid-sagittal section was evaluated by two blinded observers under ×200 light microscopy magnification. The pathological grade was scored on a scale of 0–3, as previously described, with some modification [16]. The grade criteria were as follows: 0, no lung abnormality; 1, the presence of lung injury involving <25% of the lung; 2, lesions involving 25–50% of the lung; 3, lesions involving >50% of the lung.

### Analysis of cell death

For analysis of apoptosis, we performed the terminal deoxynucleotidyl transferase dUTP nick end labeling (TUNEL) assay (Takara Biomedicals, Otsu, Japan). In brief, the sections were incubated in a mixture containing terminal deoxynucleotidyl transferase and fluorescein isothiocyanate-labeled dUTP. Following proteinase digestion to remove endogenous peroxidase, the sections were treated with peroxidase-labeled, anti-fluorescein isothiocyanate antibody. The reaction products were developed with 3, 3'-diaminobenzidine tetrahydrochloride and counterstained with methyl green. We counted the number of positive cells for TUNEL in all areas of the section under a light microscope at ×200 magnification and calculated the number of TUNEL-positive cells per field.

### Immunohistochemistry for 8-Hydroxyl-2′-deoxyguanosine (8-OHdG) and Bcl-X_L_

Following deparaffination, immunohistochemistry was performed using a modified streptavidin-biotinylated peroxidase technique with the Histofine SAB-PO kit (Nichirei Corporation, Japan). Non-specific protein staining was blocked with rabbit serum or 1% skim milk in phosphate-buffered saline (PBS) for 30 min at room temperature. The sections were incubated with mouse anti 8-OHdG monoclonal antibody (Japan Institute for the Control of Aging, NIKKEN SEIL Co., Ltd, Japan) or rabbit anti-Bcl-X_L_ antibody (Santa Cruz Biotechnology, Santa Cruz, CA) at 4°C overnight. The sections were rinsed with PBS and incubated with biotinylated anti-mouse immunoglobulin G (IgG) or biotinylated anti-rabbit IgG for 30 min. The sections were washed and treated with 0.3% hydrogen peroxide in methanol for 30 min. The slides were washed, incubated with streptavidin-biotin-peroxidase complex for 30 min, and developed according to the manufacturer's directions. In immunohistochemistry for 8-OHdG, we evaluated the degree of staining in the whole area under ×200 magnification by light microscopy. The degree of staining was graded from 0–3 according to the percentage of immunoreactive cells: 0, 0%; 1, <10%; 2, 10–50%; 3, >50%.

### Western blot analysis

The frozen lung tissues were homogenized in buffer A (25 mM HEPES, pH 7.5, 5 mM MgCl_2_, 1 mM EGTA, 1 mM phenylmethylsulfonyl fluoride, 1 μg/ml leupeptin, and 1 μg/ml aprotinin) with a Polytron homogenizer (Kinematica Co., Ltd., Switzerland). The protein concentrations of the supernatants prepared above were measured using the Bio to Rad protein assay kit (Bio-Rad Laboratories, CA, USA). Each supernatant was dissolved in sample buffer (133 mM Tris-HCl, pH 6.8, 0.1% SDS, 5% glycerol, 0.67% 2-mercaptoethanol, 1 μg/ml leupeptin and 1 μg/ml aprotinin) and boiled. One hundred twenty μg of protein were electrophoresed in each lane of an SDS-polyacrylamide gel, and the proteins were then transferred to polyvinylidene fluoride hydrophobic membranes (Millipore, Bedford, MA). The membranes were blocked with 5% nonfat dried milk in Tris-buffered saline containing 0.05% Tween-20 (TBS-T) at 4°C for 30 min. The membranes were incubated with anti-Bax (6A7) antibody (Santa Cruz Biotechnology), anti-Bcl-X_L_ antibody (Santa Cruz Biotechnology), anti-phospho p44/p42 mitogen-activated protein kinase (ERK 1/2) antibody (Cell Signaling Technology, Boston, MA), anti- p47^phox^ antibody (Santa Cruz Biotechnology), anti-p53 antibody (Santa Cruz Biotechnology), anticleaved caspase-9 antibody (Cell Signaling Technology), anti-β-tubulin antibody (Chemicon International, Temecula, CA) and anti-NF-κB-p65 antibody (Santa Cruz Biotechnology) in blocking buffer at 4°C overnight. The anti-Bax (6A7) antibody specifically reacts with the activated form of Bax, and the anti-NF-κB antibody selectivity recognizes the p65 subunit released from the activated form of NF-κB. After rinsing, the membranes were incubated with a biotinylated secondary antibody for 30 min at room temperature. The blots were developed using an enhanced chemiluminescence western blotting detection kit (Amersham Pharmacia Biotech, Buckinghamshire, UK). The membranes were scanned, and the relative intensity of the bands was quantified using Image J Ver.1.40 (National Institutes of Health, MD, USA).

### Statistical analysis

For comparison of the number of BALF cells, total protein concentration in BALF and the number of TUNEL-positive cells, ANOVA followed by Scheffe’s F test was performed. For comparison of pathological grade, Kruskal–Wallis test followed by Mann–Whitney *U* test was used. *P* < 0.05 was considered significant. Statistical analysis was performed with JMP ver.6.0.3 (SAS Institute, Cary, NC, USA).

## Results

### Probucol treatment ameliorates hyperoxic lung injury

**Fig 1:**
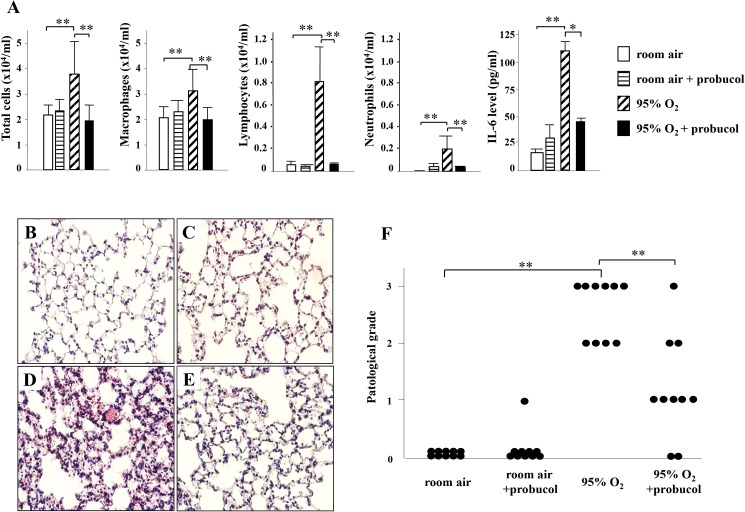
Effect of probucol on BALF and the histological findings in hyperoxic lung injury. (A) The results of cell count and level of IL-6 in BALF. Each bar shows mean ± SEM of the ten mice in each group. (B-E) Hematoxylin and eosin staining. In room-air-exposed mice without probucol treatment (B) and room-air-exposed mice treated with probucol (C), no abnormalities were observed. In the lung tissue of hyperoxia-exposed mice without probucol treatment (D), invasion of inflammatory cells, pulmonary edema, and alveolar hemorrhage were observed. In hyperoxia-exposed mice treated with probucol (E), those findings were improved. In the pathological grading, each circle corresponds to one mouse (F). Original magnifications: ×200. **P < 0.01, *p < 0.05.

The numbers of total cells, macrophages, neutrophils, and lymphocytes in BALF significantly increased in hyperoxia-exposed mice compared with those in room-air-exposed mice. Probucol treatment significantly attenuated these elevations in hyperoxia-exposed mice. Correlated with BALF findings (Fig 1A), lung tissues from room-air-exposed mice with and without probucol treatment showed no inflammation or lung injury (Fig 1C and 1B, respectively) while those from hyperoxia-exposed mice showed thickened alveolar walls infiltrated with neutrophils and lymphocytes, alveolar hemorrhage and parenchymal edema (Fig 1D). These pathological findings were ameliorated by probucol (Fig 1E and 1F). The total protein concentration in BALF significantly increased in hyperoxia-exposed mice compared with those in room-air-exposed mice. Unlike the numbers of total cells, macrophages, neutrophils, and lymphocytes, probucol treatment had little effect on the total protein concentration in BALF (S1 Fig.).

### Probucol treatment reduces oxidative stress and NADPH activity in hyperoxic lung injury

**Fig 2:**
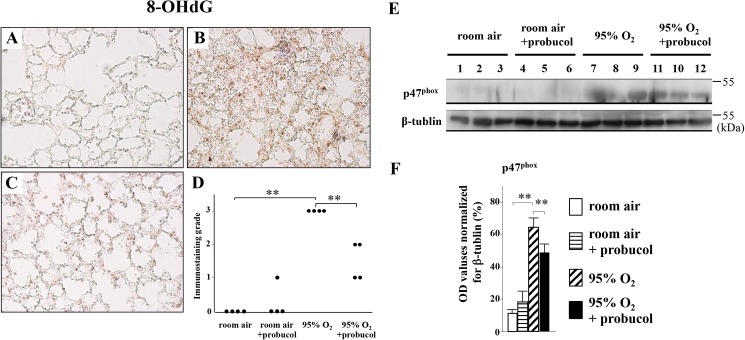
Probucol ameliorates oxidative stress and apoptosis in hyperoxic lung injury. (A-D) The results of immunohistochemistry for 8-OHdG. (A) In room-air-exposed mice treated with probucol, expression of 8-OHdG was not detected. (B) In hyperoxia-exposed mice without probucol treatment, 8-OHdG was strongly expressed in nuclei of lung epithelial cells due to the tissue damage. (C) In hyperoxia-exposed mice treated with probucol, signal of 8-OHdG was decreased. (D) The immunostaining grade for 8-OHdG was significantly decreased by probucol treatment. Original magnifications: ×200. Data are shown as the mean ± SEM from four mice in each group. (E) Western blot analysis for p47^phox^. Each lane corresponds to the data from one mouse. (F) Relative band intensities from western blot analysis. Optical density values for each individual band were normalized to β-tubulin expression from the same tissue. Data are means ± SEM from three mice. **p < 0.01.

The expression of 8-OHdG reflects oxidative stress level in the lung tissues. 8-OHdG-positive cells were not detected in lung tissues of room-air-exposed mice (Fig 2A). Positive signals for 8-OHdG increased in hyperoxia-exposed mice, and the signals were mainly located in nuclei of epithelial cells (Fig 2B). Probucol treatment significantly abrogated expression of 8-OHdG (Fig 2C and 2D). Western blot analysis showed that expression of p47^ph^°^x^, one of the components of NADPH, was significantly decreased by probucol treatment (Fig 2E and 2F).

### Probucol treatment inhibits apoptosis in hyperoxic lung injury

**Fig 3:**
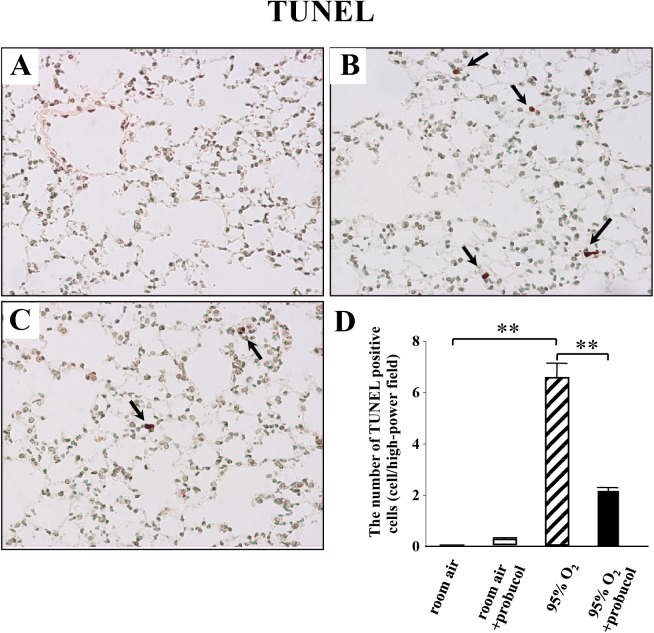
Probucol ameliorates apoptosis in hyperoxic lung injury. (A-D) The effect of probucol on TUNEL staining. (A) No positive signals for TUNEL were observed in lung tissues of room-air-exposed mice treated with probucol. (B) There were some TUNEL-positive cells (arrows in F) in the lung tissues of hyperoxia-exposed mice. (C) Probucol treatment decreased the number of TUNEL-positive cells in hyperoxia-exposed mice. (D) Quantitative result of the number of TUNEL-positive cells in lung tissues. Original magnifications: ×200. Data are shown as the mean ± SEM from four mice in each group. **p < 0.01.

As previously reported, the number of TUNEL-positive cells is correlated with apoptosis. No TUNEL-positive cells were detected in room-air-exposed mice (Fig 3A). Some bronchiolar and alveolar epithelial cells showed positive signals for TUNEL staining after hyperoxia exposure (Fig 3B). There were a few positive signals in endothelial cells. Probucol treatment significantly decreased the number of TUNEL-positive cells in lung tissues from hyperoxia-exposed mice (Fig 3C and 3D).

**Fig 4:**
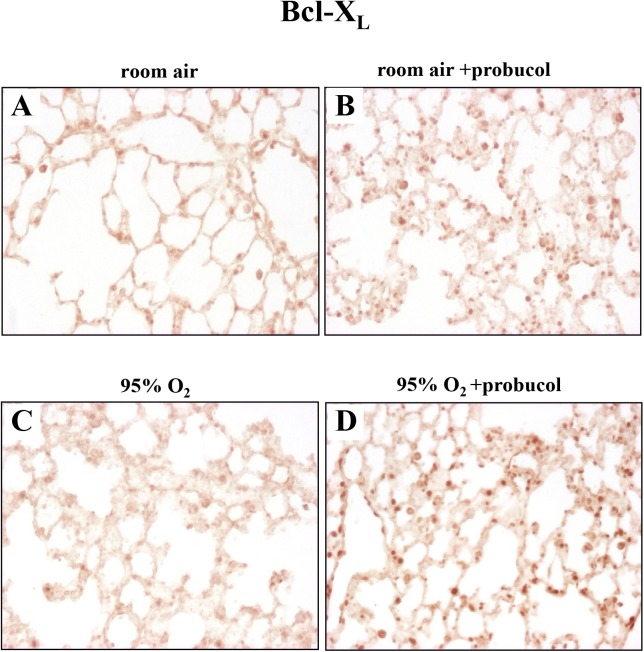
Probucol treatment increases Bcl-X_L_ expression in the lung tissues. (A) There was no detectable expression of Bcl-X_L_ in room-air-exposed mice (A). In both room-air-exposed mice and hyperoxia-exposed mice, probucol treatment increased Bcl-X_L_ expression in lung epithelial cells (B, D). In hyperoxia-exposed mice without probucol treatment, there was a low-level expression of Bcl-X_L_ (C). Original magnifications: ×200.

**Fig 5:**
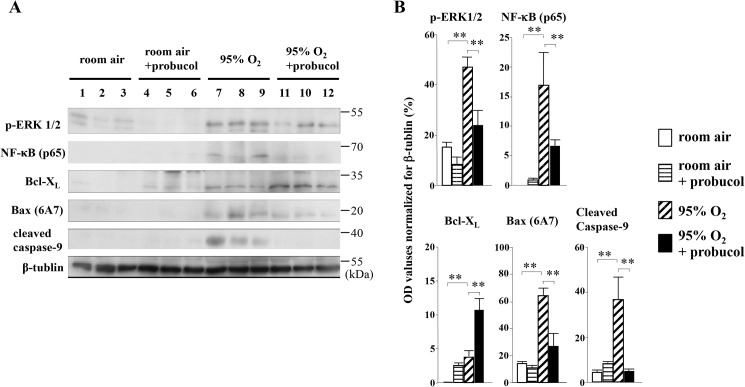
Western blot analysis for p47^phox^, phospho-ERK1/2, NF-κB (p65), Bcl-X_L_, Bax (6A7), cleaved caspase-9, and β-tubulin. (A) Each lane corresponds to the data from one mouse. (B) Relative band intensities from western blot analysis. Optical density values for each individual band were normalized to β-tubulin expression from the same tissue. Data are means ± SEM from three mice. **p < 0.01.

We then tested the expression of Bcl-X_L_, one of the apoptosis inhibitors, in lung tissues. There were weak signals for Bcl-X_L_ in lung tissues of room-air-exposed mice (Fig 4A). In hyperoxia-exposed mice without probucol treatment, Bcl-X_L_ was slightly expressed in alveolar macrophages and epithelial cells (Fig 4C). Probucol treatment enhanced Bcl-X_L_ expression in lung tissues of room-air-exposed mice, and hyperoxia-exposed mice (Fig 4B and 4D, respectively). These results were quantified by western blot analysis (Fig 5). However, Bax and cleaved caspase-9, as well as proapoptotic signals, were significantly down-regulated by probucol treatment (Fig 5). Thus, probucol treatment inhibited apoptosis in hyperoxic lung injury.

### Probucol treatment inhibits inflammation through NF-κB signaling in hyperoxic lung injury

The level of IL-6 in BALF was significantly reduced by probucol treatment (Fig 1A). Western blot analysis showed that expression of NF-κB (p65) was up-regulated in the lung tissue of hyperoxia-exposed mice compared with room-air-exposed mice (Fig 5). The band densities of NF-κB (p65) were significantly decreased by probucol treatment (Fig 5).

## Discussion

We have, for the first time, demonstrated the effects of probucol in an animal model of hyperoxic lung injury. In most studies using probucol, animals were treated with probucol by oral administration to maintain a high serum probucol concentration for a long duration, but a high systemic dose of probucol could cause detrimental side effects. In this study, mice were given probucol by the intratracheal administration because this method directly affects lung environment. Probucol treatment reduced the number of lymphocytes, neutrophils, and apoptotic cells as well the IL-6 level in BALF. Additionally, it decreased the number of apoptotic cells, 8-OHdG expression, and the pathological grade in lung tissues. As shown in our results, the intratracheal administration was highly effective and may provide a new method for probucol treatment.

The dose of probucol in the present study was 130 μg/kg. This dose was equivalent to 100 μM in 50 μl. Although determined from an *in vitro* experimental design, the concentration of probucol in a previous study was 50 μM [17]. The concentration herein was twice this concentration. In preliminary experiments, 65 μg/kg of probucol (equivalent to 50 μM) did not inhibit hyperoxia-induced lung injury (data not shown). The final concentration in the target tissue may be lower than 100 μM due to diffusion by spraying.

Although the pathogenesis of hyperoxic lung injury is not well-revealed, apoptotic signaling is considered to have an important role in hyperoxia-induced cell death, regardless of cell death phenotype [9]. There are two known apoptosis signal pathways. One is the Fas-mediated pathway, and the other is the mitochondria-dependent pathway. It is reported that hyperoxia induces apoptosis in lung epithelial cells by activation of both pathways *in vitro* [8]. In this study, expression of Bax and cleaved caspase-9 were increased in lung tissues of hyperoxia-exposed mice, which indicated that the mitochondria-dependent apoptosis pathway was activated. A recent study also suggests that role of the Fas/Fas ligand signaling pathway in the hyperoxic lung is proliferative, rather than proapoptotic [18].

Thus, the mitochondria-dependent pathway is more crucial in hyperoxia-induced epithelial apoptosis. We demonstrated that probucol treatment significantly increased Bcl-X_L_ expression and decreased Bax along with cleaved caspase-9 expression in lung tissues from hyperoxia-exposed mice. Immunohistochemistry revealed that Bcl-X_L_ was mainly expressed in lung epithelial cells from probucol-treated mice, with lesser expression in endothelial cells. Bcl-X_L_ is an antiapoptotic member of the bcl-2 family that prevents the translocation of Bax to mitochondria which lead to cytochrome c release [8]. Under oxidative stress, Bcl-X_L_ suppresses Bax activation and cell death [19]. Our results suggest that one of the antiapoptotic mechanisms of probucol may be up-regulation of Bcl-X_L_ expression in lung epithelial cell of hyperoxia-exposed mice. Moreover, it is interesting to note that probucol treatment alone tended to increase Bcl-X_L_ expression in lung tissues from room-air-exposed mice. Probucol may increase Bcl-X_L_ expression directly.

Hyperoxia causes generation of ROS, which acts as upstream of Bax activation. p47^ph^°^x^, one of the components of NADPH, is thought to be activated by hyperoxia and then induces ROS generation [20]. Umeji et al. demonstrated that probucol reduced ROS production by affecting p47^ph^°^x^ in cholesterol-fed rabbit aortas [13]. Probucol tended to decrease the expression of p47^ph^°^x^ in lung tissue from hyperoxia-exposed mice. Therefore, one of the mechanisms by which probucol suppressed oxidative damage, may be through inhibition of NADPH oxidase activation. Zhang et al. reported that NADPH oxidase was of key importance to the generation of ROS that induced lung epithelial cell death through the activation of ERK 1/2 and caspases [21]. They also demonstrated that hyperoxia-induced cell death *in vitro* and *in vivo* was attenuated by PD98059, which is an inhibitor of the mitogen-activated protein or ERK kinase ERK 1/2 pathway. There is growing evidence to suggest that ERK 1/2 activation is also involved in cell death signaling [22]. Our results showing that ERK 1/2 was activated in hyperoxia-exposed mice are compatible with previous studies.

IL-6 is a pleiotropic cytokine, whose production in inflammatory tissue is induced by NF-κB [23]. IL-6 is an optimal marker to evaluate inflammation in hyperoxic lung injury in mice [24]. In this study, both the level of IL-6 in BALF and the expression level of NF-κB in lung tissues were significantly increased in hyperoxia-exposed mice, and probucol significantly decreased the number of inflammatory cells and IL-6 levels and decreased the expression of NF-κB in lung tissue. Our results suggest that probucol reduced inflammatory reactions, in part, through the suppression of IL-6 release by inhibition of NF-κB activation. Recent evidence also showed that probucol ameliorated ischemic brain injury through inhibition of NF-κB pathway [25].

However, there are two limitations in this study. First, we did not measure other cytokines/chemokines in BALF. Second, we could not determine the effect of probucol on each constituent cell in the lung. Therefore, further studies are required.

In summary, this study shows that probucol attenuates the development of hyperoxic lung injury through antioxidant, antiapoptotic, and anti-inflammatory effects. The direct administration of probucol into the lung may represent a novel strategy for the treatment of ARDS and hyperoxic lung injury.

## Supporting information

S1 Fig Total protein concentration in bronchoalveolar fluid. Data are shown as the mean ± SEM from four mice per group. *P < 0.05.
(PPTX)
