# Supplementary material for: Probucol attenuates hyperoxia-induced lung injury in mice
Source: PLoS One. 2017 Apr 6;12(4):e0175129. doi: 10.1371/journal.pone.0175129 (PMC5383131; doi:10.1371/journal.pone.0175129)
Supplement: S1 Fig — Data are shown as the mean ± SEM from four mice per group. *P < 0.05. (PPTX) [file pone.0175129.s001.pptx]

## Slide 1
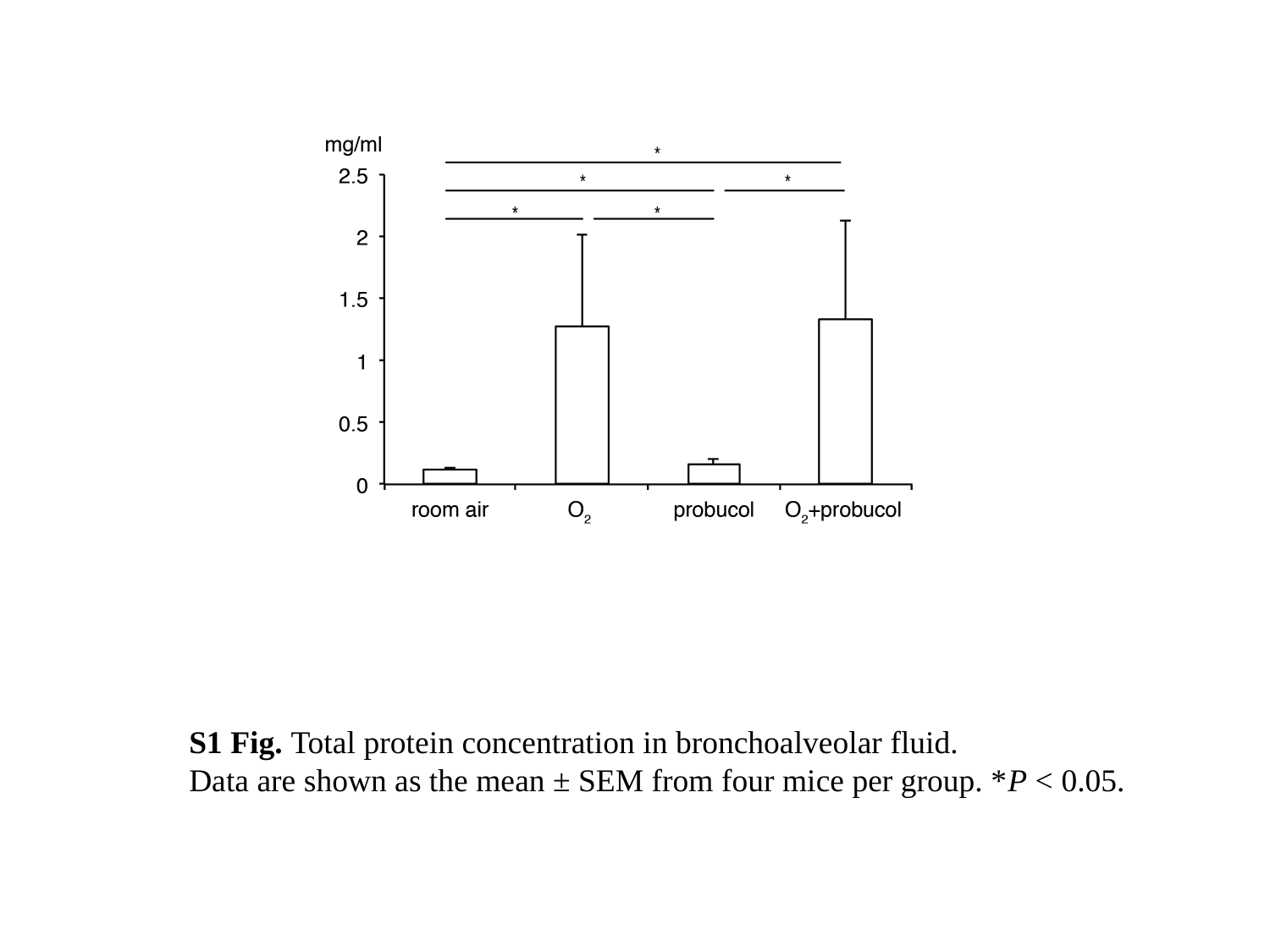

S1 Fig. Total protein concentration in bronchoalveolar fluid.
Data are shown as the mean ± SEM from four mice per group. *P < 0.05.
